# Reducing Internally Generated Demand Through Scheduled Communication Time in a Remote Salaried Family Medicine Clinic

**DOI:** 10.1177/21501319251364922

**Published:** 2025-08-19

**Authors:** Sam Seshadri, Jake Reaser, Jeffrey DC Irvine

**Affiliations:** 1 College of Medicine, University of Saskatchewan, Canada; 2 Northern Medical Services, Department of Family Medicine, College of Medicine, University of Saskatchewan, Canada

**Keywords:** appointments and schedules, continuity of patient care, quality improvement, patient preference, access to care, community health, patient-centeredness, practice management, primary care, rural health

## Abstract

**Background:**

In salaried primary care systems, internally generated demand, such as physician-initiated recalls, can limit timely access for other patients.

**Objective:**

To evaluate whether reserving protected time for unscheduled physician phone calls decreases recall appointment volume.

**Design:**

A quality improvement intervention with a before-and-after analysis.

**Setting:**

A salaried family medicine clinic in northern Saskatchewan.

**Participants:**

All clinic patients recalled for visits between October 2024 and February 2025 (1005 recalls).

**Intervention:**

A daily 30-min block was reserved in each physician’s morning schedule for discretionary phone-based follow-up.

**Main Measures:**

Total number of recalls, recall reasons, and patient demographics before and after implementation.

**Key Results:**

Recall appointments dropped by 28.7%, particularly those related to bloodwork, imaging, and specialist communication. However, the time invested in protected communication slots exceeded the time saved from reduced recalls, suggesting a shift in physician workload rather than an overall reduction.

**Conclusion:**

Allocating protected communication time may support more flexible follow-up and improve task completion efficiency, but does not appear to meaningfully expand physician appointment access.

## Introduction

Primary care systems are increasingly burdened not only by growing external patient demand but also by internally generated follow-up requests from providers, contributing to service pressure and fragmentation in care delivery.^1^ In remote or resource-limited settings, managing this demand is crucial to maintaining equitable access.^2^ These follow-up requests may not always require a full-length visit. In salaried systems, there is no financial incentive to generate unnecessary follow-up appointments. On the contrary, doing so may reduce access for new or more urgent patients in the community.

The La Ronge Medical Clinic is a salaried family medicine practice located in northern Saskatchewan, serving a catchment population of approximately 16 000 across a broad geographic area. Physicians are employed by Northern Medical Services, a division of the University of Saskatchewan, through 2 main models: regular positions, in which providers are embedded in the community with ongoing patient continuity, and itinerant contracts, where physicians work in block rotations, spending several weeks in-community followed by scheduled time away. As a publicly funded clinic, both regular and itinerant physicians are not incentivized based on patient volume. Instead, clinical priorities emphasize equitable access and continuity of care for all community members.

The Medical Clinic usually books patients every 15 min. There are 2 to 7 physicians working in the clinic on any 1 day, with a medical office assistant (MOA) ratio of approximately 0.75 assistants per physician. One physician’s schedule in the afternoon every day is left open for urgent, same-day booking. The rest of the physician’s schedules are pre-booked. Time-to-third-next-available appointment has been around 0 to 3 days for several months. Even still, as the Clinic tries to move toward true advanced access, optimizing the patient schedule and reducing internal demand is necessary.

Without sufficient MOA capacity to support outbound patient communication, the clinic implemented a 30-min morning time slot for unscheduled physician activity, starting December 2024. This time was designated primarily for short phone calls to patients, to be used at physician discretion, to reduce the need for full recall appointments. While increasing MOA support was a desired potential solution, hiring constraints within our clinic’s operational structure, which is funded through the Saskatchewan Ministry of Health, has made this alternative unattainable thus far. As such, the intervention focused on redistributing physician time within existing staffing resources. Future efforts may revisit MOA-led models if additional personnel become available.

This study evaluates whether a structural scheduling intervention of a 30-min unscheduled morning slot for discretionary physician calls could reduce recall generation by replacing full visits with brief communications, where appropriate.

## Methods

Recall data was collected through the La Ronge Medical Clinic’s MedAccess electronic medical record. In this context, a “recall” refers to an internally generated request by a physician for a patient to return to clinic for follow-up care. These are typically initiated after a visit, test result, or specialist report when the physician determines that further discussion or management is needed. These are distinct from patient-initiated appointments and are entered into the EMR as reminders for future contact and scheduling.

One collected dataset included the number of recalls generated from August 2024 to May 2025. A second dataset included every recall on patients seen between October 2024 and February 2025. Chart reviews were done on every patient chart of the second dataset by SS and JR, collecting the reason for the recall, and the physician’s name who generated the recall. This was analyzed pre- and post-intervention to assess impact. To enhance chart review consistency and reduce subjective variation, all authors met prior to data extraction to align on coding definitions and chart review criteria. During data collection, ambiguities were flagged and discussed between all authors and resolved through consensus. While formal kappa statistics were not calculated, inter-rater agreement was monitored informally throughout, with ongoing dialogue to ensure consistency.

## Results

**Figure 1.**
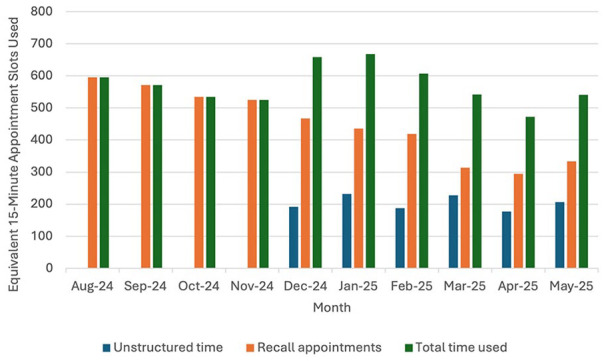
Protected call time versus recall volume and time slot use (August 2024-May 2025).

The total number of recalls generated by the La Ronge Medical Clinic physician group is depicted in Figure 1, ranging between 295 and 595 per month. The number of 15-min time slots reserved for unstructured patient phone calls ranged from 177 to 232. When combined, the total number of time slots used, either for calling or for patient recall, increased following the intervention.

One thousand and 5 recall tasks were reviewed from October 2024 to February 2025. Reasons that a physician recalled a patient are outlined in Table 1, with the percent change from before and after the implementation. Pre-implementation tasks included those from October and November 2024, while post-implementation tasks were from January and February 2025. Recalls were commonly requested to discuss test results, alter medications, and to check up on patients. Implementing an unstructured patient contact time within the physician’s schedule decreased the number of recalls especially for medication adjustments and to discuss test results, while least affecting recalls requesting a patient return for a physical exam.

**Table 1.** Reasons for Recalls.

Reason for recall	Number pre-implementation (n = 363) (%)	Number post-implementation (n = 259) (%)	% change
Test results (laboratory)	91 (25.1)	66 (25.5)	−27.5
Test results (imaging)	39 (10.7)	25 (9.7)	−35.9
Check up on patient	57 (15.7)	45 (17.4)	−21.1
Medication adjustment after results	58 (16)	40 (15.4)	−31
Other medication adjustment	13 (3.6)	7 (2.7)	−46.1
Discuss specialist report	22 (6.1)	14 (5.4)	−36.3
Physical exam request	39 (10.7)	31 (12)	−20.5

Of 26 physician providers, 13 providers saw a high recall reduction of more than 30% after the schedule implementation, 5 providers had a moderate reduction of 0% to 30%, and 8 providers showed either no reduction or an increase in recalls. To further investigate variation in recall reductions, physician providers were categorized by role and experience level (Table 2). Among the 5 groups, itinerant physicians had the highest average number of recalls prior to the intervention (mean: 19.3), followed by itinerant physicians in their first 5 years of practice (mean: 15.0). After implementation, regular physicians demonstrated the largest average reduction in recalls (–5.9 recalls; –43.5%), while regular physicians in their first 5 years saw a smaller reduction (–1.2 recalls; –9.7%). In contrast, itinerant physicians in their first 5 years of practice had a moderate reduction (–2.8 recalls; –18.9%), and itinerant physicians overall showed a substantial decrease (–7.7 recalls; –39.6%). Residents generated the fewest recalls both before and after the intervention.

**Table 2.** Provider Recall Summary by Physician Type.

Physician type	Number of providers	Mean number of recalls per provider over 2 months	Mean number of recalls per provider over 2 months	Mean change (%)
		Pre-implementation	Post-implementation	
Itinerant	3	19.33	11.67	−39.6
Itinerant first 5 years	6	15	12.17	−18.9
Regular	8	13.5	7.625	−42.5
Regular first 5 years	5	12.4	11.2	−9.7
Resident	4	4.75	2.5	−47.4

## Discussion

The 30-min discretionary time block was associated with fewer recall appointments. However, when measured against the number of time slots allocated for these calls, the reduction in visit-based recalls did not exceed or match the added phone call time. This suggests that while some visits were avoided, the overall demand on physician time was redistributed rather than reduced.

The data suggests that many recall types are particularly well-suited to brief telephone calls. This likely reflects the appropriateness of asynchronous or brief interactions for these clinical tasks. This finding aligns with previous research, including the Choosing Wisely initiative, which emphasizes avoiding low-value follow-up care when clinically safe to do so.^3^ By understanding the nature of these common recalls, we can better tailor future scheduling strategies that triage follow-ups to their most appropriate format.

Provider-level data revealed meaningful variation in both baseline recall volume and response to the intervention. Itinerant physicians generated more recalls on average prior to the intervention than their Regular counterparts, suggesting that continuity of care and familiarity with local resources may influence recall practices. Regular physicians, who may be more embedded in the community and clinical team, may be better positioned to manage follow-up needs efficiently or delegate tasks through asynchronous methods.

Physicians with more than 5 years of experience, regardless of Regular or Itinerant position, demonstrated the most consistent and substantial reductions in recall volume. Those in their first 5 years had more variable outcomes, including some increases. This may reflect differences in clinical confidence, efficiency with documentation and EMR use, or a tendency to rely more on in-person reassessment as a safety net early in practice. Although the sample sizes in these subgroups were small, this pattern reinforces the value of targeted mentorship and feedback mechanisms tailored to provider experience.

Variation may also reflect differences in clinical style, or disparities in how the protected time was used. Some physicians may have used the time for other non-recall tasks, while others fully embraced its purpose to replace in-person visits. This heterogeneity emphasizes that simple structural changes must be paired with cultural and educational support to ensure consistent uptake and long-term success of practice redesigns.^4^

While recall volume declined, the time reclaimed from reducing these appointments did not fully offset the time allocated for protected calls. Although providers may have replaced some visit-based recalls with brief calls, the total time invested in these interactions may have remained the same or even increased. This may be because physicians were unable to contact some patients despite calling multiple times, or physicians did not have any patients that needed recalling at that time. This highlights a nuanced outcome, where the intervention restructured physician workflow to potentially improve continuity and responsiveness, but did not clearly decrease time demand. The perceived benefits are therefore more likely in the realm of patient experience and system fluidity, rather than operational efficiency. These insights are critical for informing future interventions aimed at internal demand management in salaried or non-incentive-based primary care systems.

Murray and colleagues’ work on the Advanced Access model underscores the transformative potential of deliberate structural changes in primary care scheduling.^5^ These case studies showed that care coordination can be improved, and unnecessary in-person visits minimized, when clinical teams have intentional, protected time to close communication loops, particularly for results and follow-ups. Our findings align strongly with this evidence, indicating that even a modest intervention can replicate many of the outcomes achieved through larger redesign efforts. It validates the notion that improving patient care does not require new technology or staff, only thoughtful scheduling. While the principles of Advanced Access align with our structural changes, the relative impact of our intervention was more modest, suggesting that broader redesign efforts may be required to realize larger gains.

Importantly, protected time is not merely a scheduling change. Rather, it represents a cultural shift.^4^ It redefines productivity from volume of visits to quality and appropriateness of care. In salaried models like ours, this alignment is particularly critical, as the absence of fee-for-service incentives enables decisions based on need rather than billing potential. Moreover, it may reduce cognitive load on physicians by allowing task completion during designated times, rather than deferring to lunch breaks or evenings, thereby improving provider wellness and job satisfaction.

While this intervention modestly reduced the volume of in-person recall visits, its impact on patient and provider experiences remains unknown. Future research should explore how protected communication time influences perceived continuity, satisfaction, and clinical quality from both patient and provider perspectives. It is possible that while workload is redistributed, the perceived value of care may change. Understanding whether certain recall types lose therapeutic or relational value when converted to quick, non-face-to-face formats would be important. These questions are especially relevant in salaried models where efficiency gains must still align with patient-centered care. Capturing perspectives on the appropriateness of recalls, and co-developing recall criteria with clinical teams, could also help refine the intervention and ensure its sustainability.

Additional future efforts could focus on providing individualized feedback to providers regarding their recall generation patterns, for insight into how their recall practices compare to peers. This can serve as a prompt for self-reflection and voluntary change, supporting both efficiency and appropriateness in clinical follow-up. Formal tracking of how the protected communication time is used could offer richer understanding of workflow impact and inform refinement of the intervention. Additionally, the current timing may not be optimal for all physicians or patients. Future adaptations could explore relocating the time block to another time of day, such as one traditionally associated with higher clinic no-show rates. Evaluating the effectiveness of various time slots through a trial or rotation model could yield insights into balancing access, efficiency, and successful patient contact.

### Limitations

No patient or provider satisfaction data was collected, which could contextualize the perceived quality of reduced-visit care. Additionally, this evaluation did not account for whether calls during the protected time block were exclusively used to offset recall visits. The actual use of these blocks may have varied across providers, reducing the certainty that reductions in recalls were attributable solely to this intervention. This project was led by physician and student members of the clinic group, which may have influenced their interpretation of its outcomes. However, data analysis was grounded in objective clinical data extracted from the EMR and involved multidisciplinary review to mitigate bias.

## Conclusion

Protected discretionary time for unscheduled communication within physician schedules was associated with a modest reduction in recall volume. However, this came with a greater increase in protected time allocation. While this restructuring may improve continuity and responsiveness, it did not clearly reduce physician workload. In salaried, resource-limited family medicine settings, such interventions may improve care coordination but should be implemented with realistic expectations regarding efficiency.
